# Solving the woolly mammoth conundrum: amino acid ^15^N-enrichment suggests a distinct forage or habitat

**DOI:** 10.1038/srep09791

**Published:** 2015-06-09

**Authors:** Rachel Schwartz-Narbonne, Fred J. Longstaffe, Jessica Z. Metcalfe, Grant Zazula

**Affiliations:** 1 Department of Earth Sciences, The University of Western Ontario, London, ON, N6A 5B7, Canada; 2 Department of Anthropology, University of British Columbia, Vancouver, BC, V6T 1Z1, Canada; 3 Government of Yukon, Tourism & Culture (Palaeontology Program), Whitehorse, YT, Y1A 2C6, Canada

## Abstract

Understanding woolly mammoth ecology is key to understanding Pleistocene community dynamics and evaluating the roles of human hunting and climate change in late Quaternary megafaunal extinctions. Previous isotopic studies of mammoths’ diet and physiology have been hampered by the ‘mammoth conundrum’: woolly mammoths have anomalously high collagen *δ*^15^N values, which are more similar to coeval carnivores than herbivores, and which could imply a distinct diet and (or) habitat, or a physiological adaptation. We analyzed individual amino acids from collagen of adult woolly mammoths and coeval species, and discovered greater  ^15^N enrichment in source amino acids of woolly mammoths than in most other herbivores or carnivores. Woolly mammoths consumed an isotopically distinct food source, reflective of extreme aridity, dung fertilization, and (or) plant selection. This dietary signal suggests that woolly mammoths occupied a distinct habitat or forage niche relative to other Pleistocene herbivores.

Woolly mammoths (*Mammuthus primigenius*) were keystone herbivores in the Pleistocene mammoth steppe^1^,^2^. This megacontinental biome was inhabited by a now-extinct community of mammals, dominated by woolly mammoth, horse and bison. The mammoth steppe reached from north-western Canada, across the exposed Bering Isthmus, to Western Europe^3^. The ecological role of woolly mammoths within this ecosystem has been a subject of vigorous investigation^3^,^4^,^5^. Reconstructions of woolly mammoth behaviour and physiology have been largely based on morphology^4^. Isotopic studies of bulk tissues have provided independent tests of morphology-based hypotheses, as well as suggesting new ones^3^,^6^,^7^,^8^,^9^. Compound-specific isotopic studies can provide a further level of understanding of ecosystem functioning within the mammoth steppe.

Bulk collagen nitrogen isotopic compositions (*δ*^15^N_Bulk_) are commonly used in ecological studies to reveal the diet and trophic level of a species, as these values typically reflect the isotopic compositions of the plants at the base of the food web plus a 2–5‰ increase with each trophic level^10^. As a result, the *δ*^15^N_Bulk_ values of mammoth-steppe herbivore collagen are commonly ~+6‰ where the values of carnivores (~+9‰) are higher, reflective of this trophic enrichment^3^. The carnivore- rather than herbivore-like *δ*^15^N_Bulk_ values of woolly mammoth collagen (~+8‰^3^) are seemingly problematic and therefore require examination. The various hypotheses to explain this phenomenon (unique diet, niche feeding in a special habitat or distinct metabolic processes^3^,^6^,^7^,^8^,^9^,^11^,^12^,^13^,^14^) have different implications for our understanding of the now-vanished mammoth steppe ecosystem, woolly mammoth ecology, and related factors that contributed to extirpation of the woolly mammoth in this region.

Woolly mammoths may have consumed plants with higher *δ*^15^N values, such as graminoids and herbs rather than woody vegetation^7^,^12^,^13^, as suggested by the morphology of their enamel plates^4^. However, an herbaceous diet alone is not sufficient to fully explain the woolly mammoth’s high *δ*^15^N values; some further form of habitat or plant selection is also required^15^. While modern Arctic graminoids and forbs from some sites have a *δ*^15^N range of –0.3 to +10‰, the average value of these species ranges from ~+1 to ~+4‰^16^, and still other studies have reported maximum *δ*^15^N values for modern sedges of +2‰^17^ and for modern herbs of +5.3‰^18^. The majority of these plants, therefore, are not sufficiently enriched in ^15^N to explain the woolly mammoth *δ*^15^N_Bulk_ values. Plants growing in drier habitats, however, have higher *δ*^15^N values than plants from a more mesic environment^7^, and woolly mammoths may have eaten plants experiencing water-stress^3^,^8^. Water stress can also cause ^13^C-enrichment of plants^19^. This enrichment, however, is unlikely to be directly observable in woolly mammoth collagen, because its carbon isotopic composition is likely dominated by the low *δ*^13^C values of fat reserves used to survive the winter^7^.

Several other factors could also have contributed to high *δ*^15^N values for woolly mammoth collagen. Woolly mammoths that had small ranges, or repeatedly travelled the same routes, could have deposited significant quantities of faeces in those areas^12^, causing ^15^N-enrichment in plants arising from this dung fertilization^20^. Partially decayed plant material can also have higher *δ*^15^N values than the original living plant^21^. Woolly mammoths may have removed snow and ice cover by trampling and (or) with their tusks^5^, allowing them to forage on winter-killed plants generally not utilized by other large herbivores that did not share the mammoth ecological niche. It has also been proposed that woolly mammoths had distinct metabolic processes, such as increased levels of nitrogen recycling associated with winter starvation^22^,^23^ or poor quality food with low protein levels^3^,^7^,^8^,^9^,^11^,^13^,^14^,^24^, or that woolly mammoths engaged in coprophagy^12^.

The nitrogen isotopic compositions of the individual amino acids in collagen, as opposed to bulk collagen, enable discrimination between ^15^N-enrichment occurring at the base of the food chain prior to consumption (source amino acids) versus that associated with metabolic processes (trophic amino acids) (Fig. 1). Phenylalanine (Phe) and glutamate (Glu) have been identified as characteristic of source and trophic amino acids, respectively^25^,^26^. The *δ*^15^N_Phe_ value reflects the isotopic composition of those amino acids in plants at the base of the food web, while the *Δ*^15^N_Glu-Phe_ spacing (*δ*^15^N_Glu_ – *δ*^15^N_Phe_) serves as a proxy for metabolic enrichment of ^15^N in the consumer’s body^25^.

## Results

Eight Pleistocene megafauna species were analyzed in this study. These included four herbivore species: woolly mammoth (*Mammuthus primigenius*), mastodon (*Mammut americanum*), horse (*Equus* sp.) and giant beaver (*Castoroides ohioensis*), and four carnivore species: brown bear (*Ursus arctos*), scimitar cat (*Homotherium serum*), wolf (*Canis lupus*), and short-faced bear (*Arctodus simus*) (see Supplementary Table S1). All samples were obtained from specimens collected near Old Crow, Yukon, Canada (latitude: 67°34’N; longitude: 139°48’W). A subset of these specimens was dated, including both herbivores and carnivores. Two horse specimens were dated to 18,370 and 27,180 ^14^C years BP. The rest of the specimens yielded effectively non-finite radiocarbon dates ≥37,200 ^14^C yr BP, and one specimen was dated by context to ~140,000 years BP (see Supplementary Table S1)^27^,^28^. All collagen samples were considered well preserved based on their collagen yields, C/N ratios, and carbon and nitrogen contents^29^ (see Supplementary Table S2).

**Figure 2:**
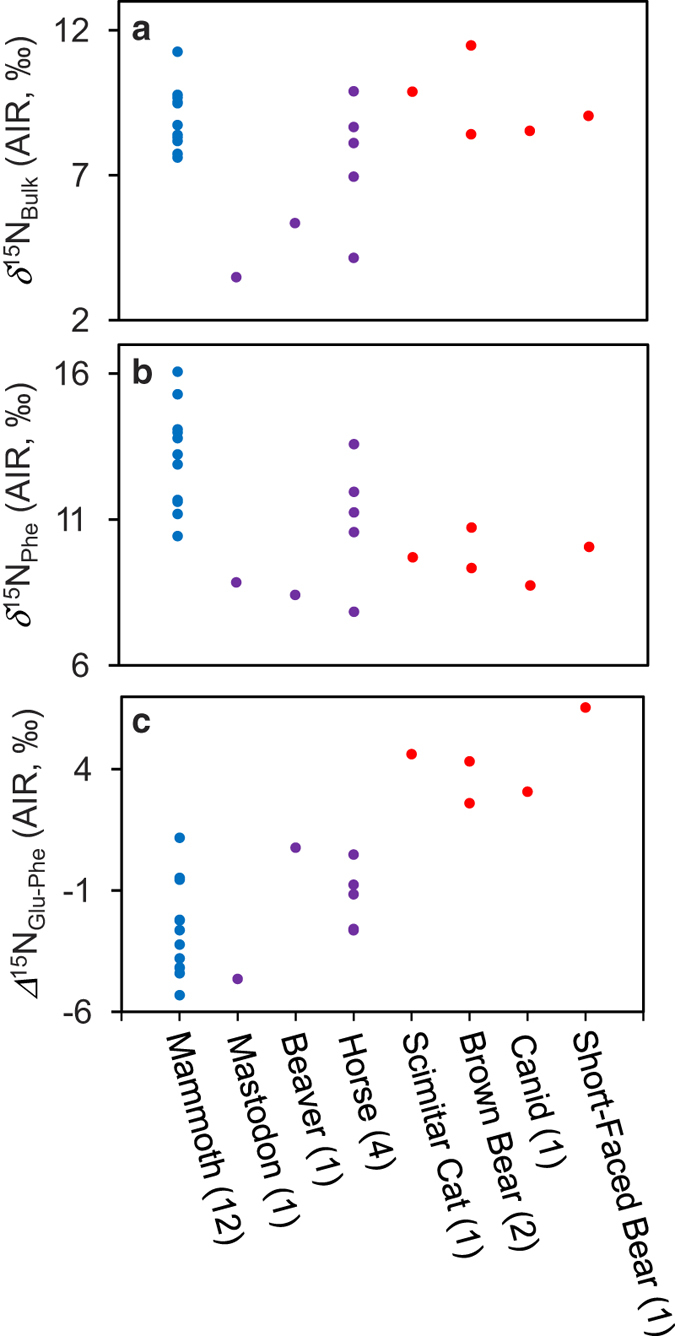
Nitrogen isotopic compositions of Pleistocene Old Crow megafauna. (**a**) Bulk collagen nitrogen isotopic compositions (*δ*^15^N_Bulk_). Results for woolly mammoths are displayed in blue, other herbivores in purple, and carnivores in red. (**b**) Phenyalanine (source) amino acid nitrogen isotopic compositions (*δ*^15^N_Phe_) of each species. (**c**) Difference between the nitrogen isotopic composition of glutamate and phenylalanine (*Δ*^15^N_Glu-Phe_) for each species.

The *δ*^15^N_Bulk_ values for the Old Crow samples follow the pattern previously observed for Pleistocene megafauna^3^,^8^,^11^; woolly mammoth collagen generally has *δ*^15^N_Bulk_ values similar to the carnivores and higher than the other herbivores, with some overlap with horses (Fig. 2a). The *δ*^15^N_Phe_ values of woolly mammoth collagen, however, are higher than those of the carnivores and most of the other herbivores (Fig. 2b); horses with high *δ*^15^N_Bulk_ values for collagen show the most overlap with the *δ*^15^N_Phe_ values of woolly mammoths. Woolly mammoth *Δ*^15^N_Glu-Phe_ spacings overlap those of the other herbivores but are lower than the *Δ*^15^N_Glu-Phe_ spacings of the carnivores, extending to negative values for most samples (Fig. 2c). Negative *Δ*^15^N_Glu-Phe_ spacings have observed in terrestrial herbivores previously^25^,^30^ and may be the result of relatively high *δ*^15^N_Phe_ values in vascular plants^31^.

## Discussion

The high *δ*^15^N_Phe_ values of the woolly mammoth imply that they selectively consumed plants more enriched in ^15^N than forage consumed by most of the other herbivores. The fact that the *δ*^15^N_Phe_ values of woolly mammoths are higher than those of carnivores suggests that the latter consumed herbivores subsisting on less ^15^N-rich forage than consumed by woolly mammoths. In short, the carnivores did not consume significant quantities of woolly mammoth. Partial overlap between horse and woolly mammoth *δ*^15^N_Bulk_ and *δ*^15^N_Phe_ values likely indicates that horses exploited a similar niche to the woolly mammoth in some cases. The low *Δ*^15^N_Glu-Phe_ spacings of woolly mammoths indicate that their *δ*^15^N_Bulk_ values arise from the higher *δ*^15^N values of the plants they consumed, and not from a specialized metabolic process.

It seems that woolly mammoths occupied a specialized dietary or habitat niche. A dietary niche implies that woolly mammoths selected particular herbaceous plants or consumed large quantities of decayed plants in winter, while a habitat niche suggests that woolly mammoths occupied more arid habitats, or lived in distinct ranges where they left considerable quantities of dung that fertilized the plants growing there. While some Old Crow horses appear also to have exploited such a niche, it was not generally shared by other mammoth steppe megafauna. The Old Crow samples likely represent various time intervals through the late Pleistocene and potentially varied climate regimes. The fact that the relative differences in average *δ*^15^N_Bulk_ values among herbivore species are generally consistent across the mammoth steppe^3^ suggests that most herbivore species ate the same forage types regardless of climatic differences or time period. This implies that mammoth steppe herbivores targeted specific forage types.

Two significant conclusions arise from these observations. First, the woolly mammoth occupied a distinct niche from other contemporaneous herbivores. This unique habitat or forage existed across the entirety of the mammoth steppe, although its size may have varied with changing climate across geographic or temporal zones. Other evidence of the woolly mammoth’s dependence on a specialized niche may be provided by the retraction of woolly mammoth populations into small, isolated refugia during the last interglacial warm period (MIS 5e, 130-116 kyr BP), and the subsequent re-expansion upon return to glacial conditions^32^. An investigation of the isotopic compositions of woolly mammoths from a variety of time periods and sites across the mammoth steppe could reveal the extent of adaptability of the woolly mammoth to disruptions in its niche, such as may have occurred with the onset of climatic shifts at the end of the Pleistocene^33^.

Second, the horse *δ*^15^N_Bulk_ and *δ*^15^N_Phe_ values overlap those of the woolly mammoth and the other herbivores. This implies that horses fed from a wide diversity of habitats or forage types, including the woolly mammoths’ niche. Such behaviour would suggest that the mammoth steppe ecosystem supported herbivores with a variety of ecological adaptations, and that even in the Pleistocene Arctic, resources were sufficiently abundant to support both specialist and generalist strategies.

## Methods

### Radiocarbon dating

Radiocarbon dates for six woolly mammoths discussed here have been published previously^27^,^28^. A further subset of samples was dated for this study; these included two other herbivores and two carnivores (see Supplementary Table S1). Collagen was extracted, combusted, graphitized and radiocarbon dated at the University of Arizona Accelerator Mass Spectrometry (AMS) Laboratory. All dates are presented as uncalibrated radiocarbon years before present (mean ±1SD).

### Bulk collagen nitrogen isotope (*δ* ^15^N_Bulk_) analysis

Collagen for *δ*^15^N_Bulk_ analysis was extracted at the Laboratory for Stable Isotope Science (LSIS) following previously published methods^28^, or was previously extracted and analyzed for another study^27^,^28^ (see Supplementary Table S2). The *δ*^15^N_Bulk_ values were obtained using a Costech Elemental Combustion System (ECS 4010) attached to a ThermoFisher Delta Plus XL IRMS or to a ThermoFisher Delta V Plus IRMS. The *δ*^15^N_Bulk_ values were measured over three analytical sessions. In the first two analytical sessions, the *δ*^15^N values were calibrated to AIR using USGS40 (L-glumatic acid; accepted value *–*4.52‰^34^) and IAEA-N2 (ammonium sulfate; accepted value +20.3‰^35^), while the third analytical session substituted USGS41 (L-glumatic acid; accepted value +47.57‰^34^) for IAEA-N2. The same standards were used to create calibration curves for determining carbon and nitrogen contents (wt%) of each sample, from which C/N ratios were calculated. Keratin (MP Biomedicals Inc., Cat No. 90211, Lot No. 9966H) was used as an internal standard in each analytical session. For a total of 18 keratin measurements over the three analytical sessions, average values (mean ±1 SD) were *δ*^15^N = +6.4 ± 0.2‰ (accepted value = +6.4‰), and C/N = 3.6 ± 0.4 (accepted value = 3.7). The standard deviation of a sample analyzed as an instrumental duplicate was *δ*^15^N_Bulk_ = ±0.0‰, and C/N = ±0.1 (1 SD). The standard deviations (1 SD) for method duplicates of *δ*^15^N_Bulk_ values ranged from 0.0 to 0.2‰, and for C/N ratios, from 0.0 to 0.3. All samples were considered to be well preserved based on their extraction yields, C/N ratios, and carbon and nitrogen contents^29^,^36^. Eight samples had high carbon and/or nitrogen contents, but this anomaly likely arises from a weighing error as they were well preserved by other measures.

### Amino acid nitrogen isotope (*δ* ^15^N_Amino Acid_) Analysis

Using collagen first extracted for *δ*^15^N_Bulk_ measurements, amino acids were hydrolysed, derivatized to their N-acetyl-methyl ester derivative, and their individual δ^15^N_Amino Acid_ values measured using an Aligent 6890N-ThermoFisher Gas Chromatograph-Combustion 3-ThermoFisher Delta Plus XL IRMS. An Agilent Technologies VF-23MS column was used in the GC. We followed published methods^25^,^37^ with only slight modifications: (i) the quantity of collagen hydrolysed was increased from 2 to 6 mg, and the quantity derivatized was increased from 0.25 to 1.5 mg; and (ii) the initial GC column temperature was set at 60 °C instead of 40 °C, and its final temperature of 250 °C was held for 15 min instead of 20 min. A representative chromatogram is shown in Supplementary Fig. S1. Three reference gas pulses were introduced into the IRMS at the beginning of each analytical session and one pulse was introduced at the end of each session. The isotopic composition of the reference gas was calibrated using four amino acid standards. Three of these standards, alanine, leucine and phenylalanine, were purchased as their NACME derivative from Sigma Aldrich. The fourth, proline, was purchased as an amino acid and derivatized in-house. The nitrogen isotopic compositions of the derivatized standards were established by multiple measurements performed in the same manner as used for isotopic analysis of bulk collagen, and calibration to AIR using international standards. The amino acid reference standards were injected every three to five runs. All samples were analyzed a minimum of three times, and the average variation was ±0.7‰ (1 SD) for phenyalanine, and ±0.7‰ (1 SD) for glutamate, with a range of 0.0–3.9‰. An internal standard, norleucine, was also analyzed. Its nitrogen isotopic compositions were offset from expected values by an average of +1.3‰.

## Additional Information

**How to cite this article**: Schwartz-Narbonne, R. *et al*. Solving the woolly mammoth conundrum: amino acid ^15^N-enrichment suggests a distinct forage or habitat. *Sci. Rep.* **5**, 09791; doi: 10.1038/srep09791 (2015).

## Supplementary Material

Supplementary Information

## Figures and Tables

**Figure 1:**
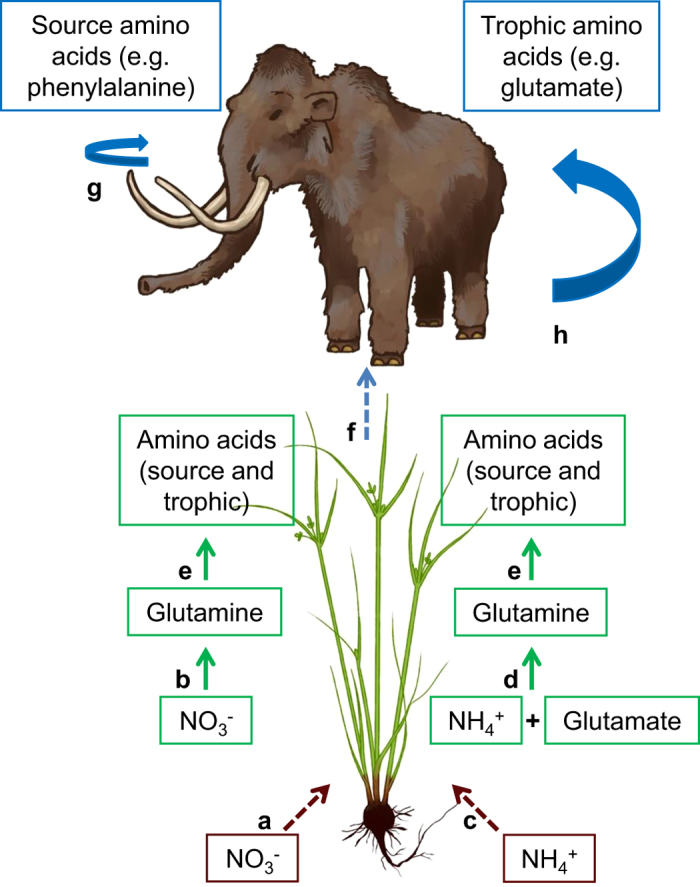
Simplified pathway for nitrogen incorporation from soil to animal protein. Arrows represent: uptake (dashed line), chemical transformations (solid line), and metabolic processes (solid curves). (**a**) Plant NO_3_^–^ uptake. (**b**) NO_3_^–^ converted to glutamine^31^. (**c**) NH_4_^+^ uptake. (**d**) **N**H_4_^+^ converted to glutamine by attachment to glutamate^31^. (**e**) Glutamine supplies nitrogen for synthesis of other amino acids. The associated shift in *δ*^15^N values depends on the specific amino acid, plant part and plant type^31^. (**f**) Consumption of amino acids by the animal. (**g**) Source amino acids are minimally involved in metabolic processes, undergoing small changes in *δ*^15^N values (e.g. increases in *δ*^15^N_Phe_ values from diet to consumer tissue are commonly ≤ 2‰^20^). (**h**) Trophic amino acids are heavily involved in metabolic processes, undergoing enrichment in ^15^N (e.g. increases in *δ*^15^N_Glu_ values from diet to consumer tissue are commonly 6–7‰^20^). Katherine Allan drew the images of grass and mammoth in Figure 1.
