# Supplementary material for: Solving the woolly mammoth conundrum: amino acid 15N-enrichment suggests a distinct forage or habitat
Source: Sci Rep. 2015 Jun 9;5:9791. doi: 10.1038/srep09791 (PMC4460640; doi:10.1038/srep09791)
Supplement: Supplementary Information [file srep09791-s1.pdf]

Supplementary Information

Solving the woolly mammoth conundrum: amino acid ^{15}N-enrichment suggests a distinct forage or habitat

Schwartz-Narbonne R, Longstaffe FJ, Metcalfe JZ, Zazula, G

Lab ID	Common Name	Smpl. No.	^{14}C Date	Latitude	Longitude	Collection Site	Tissue	Tissue Type	^{14}C Lab No.	Source
YT1RD	Mammoth	291.1	>41,100	67º58.166' N	139º33.689' W	Old Crow River	Root dentin	LRM6	AA84987*	YG
YT2RD	Mammoth	122.2	>41,100	68º03'33" N	139º46'07" W	CRH 94 Old Crow River	Root dentin	ULM6	AA84992*	YG
YT3D	Mammoth	173.5		67º29'00" N	139º55'00" W	Ch'ijee's Bluff, Porcupine River	Crown dentin	U M6		YG
YT4B	Mammoth	285.1	>39,100	67º55.1' N	139º40.7' W	OCR, REM 78-1	Bone		AA85002*	YG
YT5RD	Mammoth	60.2		68º08'44" N	139º58'12" W	OCR, Bluffs-R bank	Root dentin	LRM5/M6		YG
YT6C	Mammoth	57.1		68º11'19" N	140º32'04" W	OCR, Bluffs-R bank	Cementum	LRM6		YG
YT7B	Mammoth	325.22	>40,100	67º51.892' N	139º48.075' W	HH-68-10 Old Crow River	Bone		AA84984*	YG
YT9C	Mammoth	284.4		67º49'29" N	139º50'11" W	CRH 11 Old Crow River	Cementum	ULM6		YG
YT10 RD	Mammoth	252.2	>40,000	68º01'46" N	139º34'15" W	CRH 20 Old Crow River	Root dentin	LLM6	AA85001*	YG
YT11C	Mammoth	173.1		67º29'00" N	139º55'00" W	Ch'ijee's Bluff, Porcupine River	Cementum	U M6		YG
YT11 RD							Root dentin	U M6		
YT51T	Mammoth	317.51	MIS5, ~140,000			CRH 11 Old Crow River	Tusk dentin	Tusk	†	YG
YT68	Brown Bear	282.38		68º01'45" N	139º34'24" W	Old Crow River	Bone			YG
YT81	Short-Faced Bear	177.49		67º49'00" N	139º54'15" W	CRH 11A Old Crow River	Bone	Innominate		YG
YT82	Scimitar Cat	236.133	>37,200	68º02'12" N	139º34'40" W	CRH 67 Old Crow River	Bone		**Beta 227532**	YG
YT84	Brown Bear	236.16		68º02'12" N	139º34'40" W	CRH 67 Old Crow River	Bone			YG
AMNH3	Canid	F:AM 97114	**40,800 ± 3,900**			Old Crow River	Bone		**AA97954**	AMNH
YT129	Horse	178.9	**27,180 ± 420**	67º30'00" N	139º57'26" W	Porcupine River, downstream from Ch'ijee's Bluff	Bone		**AA103890**	YG
YT130	Horse	179.14		68º15'49" N	140º22'25" W	CRH 47 Old Crow River	Bone			YG
YT131	Horse	236.24	**18,370 ± 260**	68º02'12" N	139º34'40" W	CRH 67 Old Crow River	Bone		**AA103835**	YG
YT132	Horse	295.2	**>41,100**	67º54'25" N	139º40'57" W	HH-68-21 Old Crow River	Bone		**AA103836**	YG
YT133	Horse	315.1		68º12'48" N	140º00'41" W	CRH 44 Old Crow River	Bone			YG
AMNH1	Giant Beaver	F:AM 65186				Old Crow River	Bone			AMNH
YT8D	Mastodon	357.1	>41,100			Old Crow River	Crown dentin	M5/M6	AA84995*	YG

Supplementary Table S1: Sample information.
YG = Yukon Government, AMNH = American Museum of Natural History. Radiocarbon dates shown in bold were measured as part of this study. *Radiocarbon date previously reported[1]. † Date previously reported[2].

Lab ID	Source	Species	$\delta^{15}N_{Bulk}$	% Yield	%C	%N	C/N	$\delta^{15}N_{Phe}$	$\delta^{15}N_{Glu}$	$\Delta^{15}N_{Glu-Phe}$
YT1RD	YG	Mammoth*	+8.7	14.4	41.6	15.6	3.1	+11.6 ± 0.7	+7.2 ± 0.1	–4.4 ± 0.9
YT2RD	YG	Mammoth*	+8.4	11.0	39.2	14.6	3.1	+16.1 ± 0.9	+11.9 ± 0.5	–4.2 ± 1.4
YT3D	YG	Mammoth*	+9.7	>6.7	44.0	16.4	3.1	+15.3 ± 0.7	+11.5 ± 0.3	–3.8 ± 1.0
YT4B	YG	Mammoth*	+9.5	13.1	41.1	15.2	3.1	+14.0 ± 0.7	+10.8 ± 0.2	–3.2 ± 0.9
YT5RD	YG	Mammoth*	+9.8	11.2	42.9	15.5	3.2	+13.2 ± 0.9	+10.6 ± 0.4	–2.6 ± 1.4
YT6C	YG	Mammoth*	+7.7	10.1	42.5	15.2	3.3	+13.8 ± 1.4	+9.6 ± 1.5	–4.2 ± 2.9
YT7B	YG	Mammoth*	+7.6	15.7	42.3	15.8	3.1	+11.7 ± 0.3	+9.5 ± 0.7	–2.2 ± 1.0
YT9C	YG	Mammoth*	+8.2	7.1	41.4	15.0	3.2	+11.6 ± 0.8	+9.4 ± 0.5	–2.2 ± 1.4
YT10RD	YG	Mammoth*	+9.8	11.4	38.9	14.4	3.2	+11.2 ± 0.2	+10.6 ± 0.8	–0.6 ± 0.9
YT11C	YG	Mammoth*	+8.3	18.2	44.1	15.9	3.2	+10.4 ± 0.5	+11.6 ± 0.3	+1.2 ± 0.7
YT11RD	YG	Mammoth†	+9.5	14.3	47.2	17.6	3.1	+14.1 ± 1.3	+8.8 ± 3.9	–5.3 ± 5.2
YT51T	YG	Mammoth*	+11.3	18.6	37.9	13.9	3.2	+12.9 ± 1.3	+12.4 ± 1.0	–0.5 ± 2.2
YT68	YG	Brown Bear	+8.4	9.7	45.4	16.8	3.1	+9.3 ± 0.7	+13.7 ± 0.6	+4.4 ± 1.3
YT81	YG	Short-Faced Bear	+9.1	7.6	45.0	16.7	3.2	+10.1 ± 0.4	+16.6 ± 0.4	+6.5 ± 0.7
YT82	YG	Scimitar Cat	+9.9	8.0	46.0	17.2	3.1	+9.7 ± 0.8	+14.3 ± 0.6	+4.6 ± 1.4
YT84	YG	Brown Bear	+11.5	6.2	43.6	16.1	3.2	+10.7 ± 0.3	+13.3 ± 0.8	+2.6 ± 1.1
AMNH3	AMNH	Canid	+8.5	8.5	42.0	15.1	3.2	+8.7 ± 0.9	+11.8 ± 0.7	+3.1 ± 1.6
YT129	YG	Horse	+7.0	10.2	39.7	15.5	3.3	+11.2 ± 0.5	+8.6 ± 0.8	–2.6 ± 1.3
YT130	YG	Horse	+8.1	11.2	40.3	15.6	3.2	+10.6 ± 0.7	+11.1 ± 0.7	+0.5 ± 1.3
YT131	YG	Horse	**+9.9**	**5.4**	**36.1**	**14.0**	**3.4**	+12.0 ± 0.0	+11.2 ± 0.7	–0.8 ± 0.7
YT132	YG	Horse	+4.2	8.2	38.5	14.8	3.1	+7.8 ± 0.7	+6.7 ± 0.8	–1.1 ± 1.6
YT133	YG	Horse	**+8.7**	10.9	**42.0**	**16.2**	**3.2**	+13.6 ± 0.2	+11.0 ± 0.3	–2.6 ± 0.6
AMNH1	AMNH	Giant Beaver	+5.4	5.1	37.5	13.5	3.2	+8.4 ± 0.2	+9.2 ± 0.2	+0.8 ± 0.4
YT8D	YG	Mastodon†	+3.5	17.8	42.3	16.0	3.1	+8.9 ± 1.5	+4.2 ± 0.1	–4.7 ± 1.6

Supplementary Table S2: Nitrogen isotopic data and preservation information.
Tissue: RD = root dentin, D = crown dentin, T = tusk dentin, B = bone, C = cementum, E = enamel. The average results of duplicate measurements for bulk collagen are shown in bold-faced font. *Data for bulk collagen previously reported[1]. †Data for bulk collagen previously reported[2]. Amino acid $\delta^{15}N$ values represent the average (± 1 SD) of triplicate analyses.

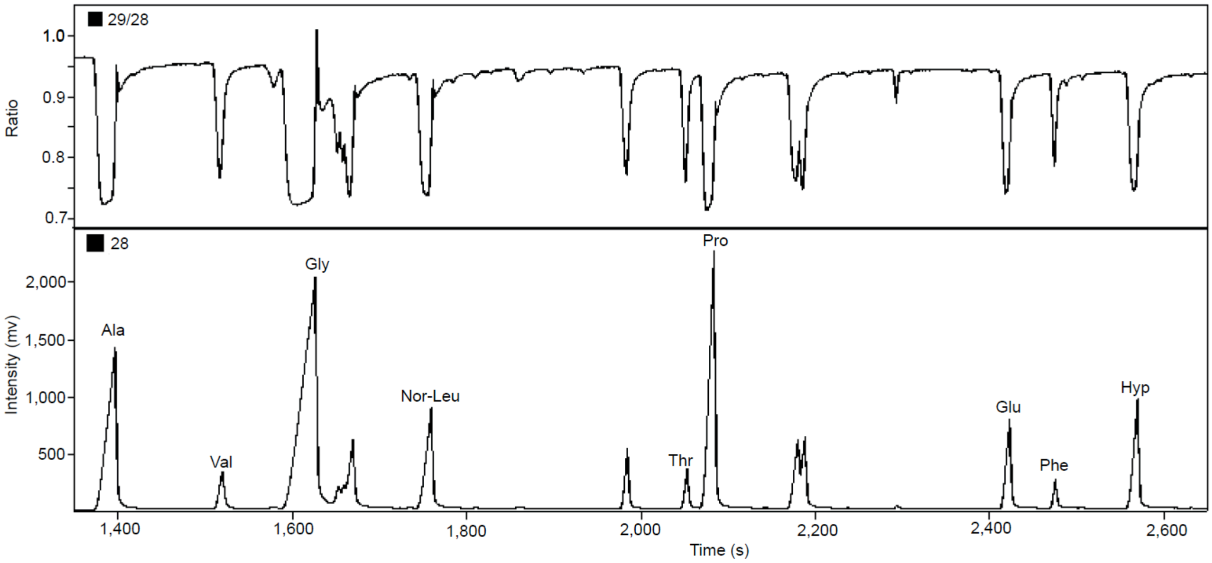

Supplementary Figure S1: Typical gas chromatogram (sample YT51T) of N-acetyl-methyl ester derivatized amino acids from collagen. Ala = alanine, Val = valine, Gly = glycine, Leu = leucine, Thr = threonine, Pro = proline, Asp = aspartate, Ser = serine, Glu = glutamate, Phe = phenylalanine and Hyp = hydroxyproline. The top scan is the ratio of mass 29/28, and the bottom scan is the amplitude of mass 28.

Supplementary References

1. Metcalfe, J. Z., Longstaffe, F. J. & Zazula, G. D. Nursing, weaning, and tooth development in woolly mammoths from Old Crow, Yukon, Canada: Implications for Pleistocene extinctions. *Palaeogeogr. Palaeoclimatol. Palaeoecol.* **298,** 257–270 (2010).

2. Metcalfe, J. Z. Late Pleistocene climate and proboscidean paleoecology in North America: Insights from stable isotope compositions of skeletal remains. Doctoral Thesis. *The University of Western Ontario.* 277 (2011).
